# NAC1 Regulates PCK2 Expression and Activates Truncated Gluconeogenesis for Growth Advantage in Ovarian Cancer Cells

**DOI:** 10.3390/ijms26199379

**Published:** 2025-09-25

**Authors:** Naomi Nakayama, Kentaro Nakayama, Puja Dey, Sultana Razia, Satoru Kyo

**Affiliations:** 1 Department of General Medicine, East Medical Center, School of Medicine, Nagoya City University, Nagoya 464-8547, Japan; 2 Department of Obstetrics and Gynecology, East Medical Center, School of Medicine, Nagoya City University, Nagoya 464-8547, Japan; 3 Department of Obstetrics and Gynecology, School of Medicine, Shimane University, Izumo 693-0021, Japan; puja1001066@gmail.com (P.D.); sultana@med.shimane-u.ac.jp (S.R.); satoruky@med.shimane-u.ac.jp (S.K.)

**Keywords:** ovarian cancer, cancer metabolism, gluconeogenesis, NAC1, PCK2, serine synthesis

## Abstract

Nucleus accumbens-associated protein 1 (NAC1), a cancer-related transcriptional regulator, is overexpressed in several malignancies, including ovarian cancer. However, its role in ovarian carcinogenesis remains unclear. We aimed to investigate whether NAC1 contributes to metabolic adaptation in endometriosis-related ovarian neoplasms (ERONs) and elucidate its regulatory mechanisms. The clinical relationship between NAC1 and its potential downstream target, phosphoenolpyruvate carboxykinase isoform 2 (PCK2), was examined using immunohistochemical analysis of ovarian cancer specimens. A cell viability assay was performed to clarify the impact of PCK2 on ovarian cancer cell viability. Reporter and chromatin immunoprecipitation (ChIP) assays were conducted to evaluate transcriptional regulation by NAC1. Metabolomic profiling was performed to assess the functional impact of the NAC1–PCK2 axis. A positive correlation between NAC1 and PCK2 expression was observed, and co-expression was associated with poor long-term survival. Knockdown of PCK2 led to a significant reduction in cell viability, indicating that PCK2 is required for maintaining cell survival. Reporter and ChIP assays confirmed that NAC1 directly binds to the PCK2 promoter via the CATG motif. The metabolomic analysis demonstrated that NAC1 promotes truncated gluconeogenesis and de novo serine synthesis through PCK2 upregulation. These findings suggest that NAC1 contributes to ovarian cancer progression by promoting metabolic adaptation, highlighting the NAC1–PCK2 axis as a potential therapeutic target for ERONs.

## 1. Introduction

Ovarian cancer is the most lethal gynecological malignancy worldwide [1], and its incidence has increased markedly over the past decade. In >70% of cases, the tumor has already disseminated outside of the ovaries when the diagnosis is made. In such cases, a combination of surgery and chemotherapy is required. First-line chemotherapy, typically involving platinum-based agents and taxanes, yields an initial response rate of over 80%; however, most patients eventually relapse. Recurrent tumors are often platinum-resistant and fatal in most women. Therefore, the development of drugs targeting specific molecular pathways could offer significant benefits to individuals affected by this devastating disease. Endometriosis-related ovarian neoplasms (ERONs) are a subset of ovarian cancers that arise from benign endometriotic cysts and exhibit distinct histopathological and molecular features, in contrast to high-grade serous carcinomas, which originate from the fallopian tube epithelium [2]. ERONs tend to show platinum resistance and are associated with a worse prognosis, particularly in advanced or recurrent stages. Consequently, targeted therapies addressing the molecular mechanisms underlying ERON development are urgently needed. Understanding the genetic and molecular pathways involved in ERON carcinogenesis is essential for the rational design of such therapeutic agents [3].

Nucleus accumbens-associated protein 1 (NAC1) is a member of the BTB/POZ family of proteins and acts as a transcriptional regulator. It plays a crucial role in the self-renewal and maintenance of pluripotency in embryonic stem cells [4]. In cancer research, NAC1 was first identified as a cancer-related transcription factor in ovarian cancer [5]. Since then, it has been found to be significantly overexpressed in several other malignancies, including colorectal, breast, renal cell, cervical, and pancreatic carcinomas, where it is associated with tumor growth and survival, early recurrence, and increased resistance to chemotherapy [6,7,8]. These findings suggest that NAC1 plays diverse roles in cancer development and may serve as a potential therapeutic target.

Recent studies have highlighted the importance of metabolic reprogramming in cancer, wherein tumor cells alter nutrient metabolism to gain a proliferative advantage. This adaptation enables efficient utilization of available resources to support rapid growth. Oncogenic signaling pathways, often activated by mutations or gene amplifications, are key drivers of these metabolic changes. Some cancer cells become dependent on specific metabolic pathways for survival, and therapies targeting these altered pathways have attracted increasing attention in cancer research. Regarding reports that NAC1 is involved in the regulation of specific metabolic pathways, we previously discovered and reported that NAC1 activated lipid metabolism by activating fatty acid synthase (FASN) expression in ovarian clear cell carcinomas (OCCCs), which were classified as one of the ERONs [9].

To understand how NAC1 contributes to cancer development, we investigated its downstream target genes. We performed microarray-based mRNA expression profiling to identify differentially expressed genes in cells with and without NAC1 knockdown. Given the growing interest in cancer metabolism and the possibility that NAC1 may regulate metabolic pathways in cancer cells, we focused on genes related to nutrient metabolism. Among the upregulated genes, we identified phosphoenolpyruvate carboxykinase isoform 2 (PCK2), a key enzyme in gluconeogenesis. Gluconeogenesis is a glucose synthesis pathway that converts non-carbohydrate precursors into glucose, primarily in hepatic and renal tissues. It is activated under glucose-deprived conditions to maintain systemic glucose homeostasis. During this process, amino acids and other non-carbohydrate substrates are converted into oxaloacetate (OAA) through the tricarboxylic acid (TCA) cycle. PCK2 catalyzes the conversion of OAA into phosphoenolpyruvate (PEP), a critical step in the gluconeogenic pathway [10]. Subsequently, PEP is converted into glucose through the remaining steps of gluconeogenesis. Recent evidence has shown that PCK2 is overexpressed in several cancer types and plays a key role in cancer cell growth and survival by promoting metabolic adaptation [11]. Although approximately 25% of ovarian cancers exhibit elevated PCK2 expression, only 1.4% harbor genetic alterations, such as gene amplification or mutation, according to data from The Cancer Genome Atlas [12]. Based on these findings, we aimed to investigate the regulatory mechanism by which NAC1 controls PCK2 expression and elucidates its role in ERON development. Additionally, we explored how the NAC1–PCK2 axis influences metabolic pathways involved in ovarian cancer progression.

## 2. Results

### 2.1. Expression Levels of NAC1 and PCK2 Are Positively Correlated in ERONs

**Figure 1:**
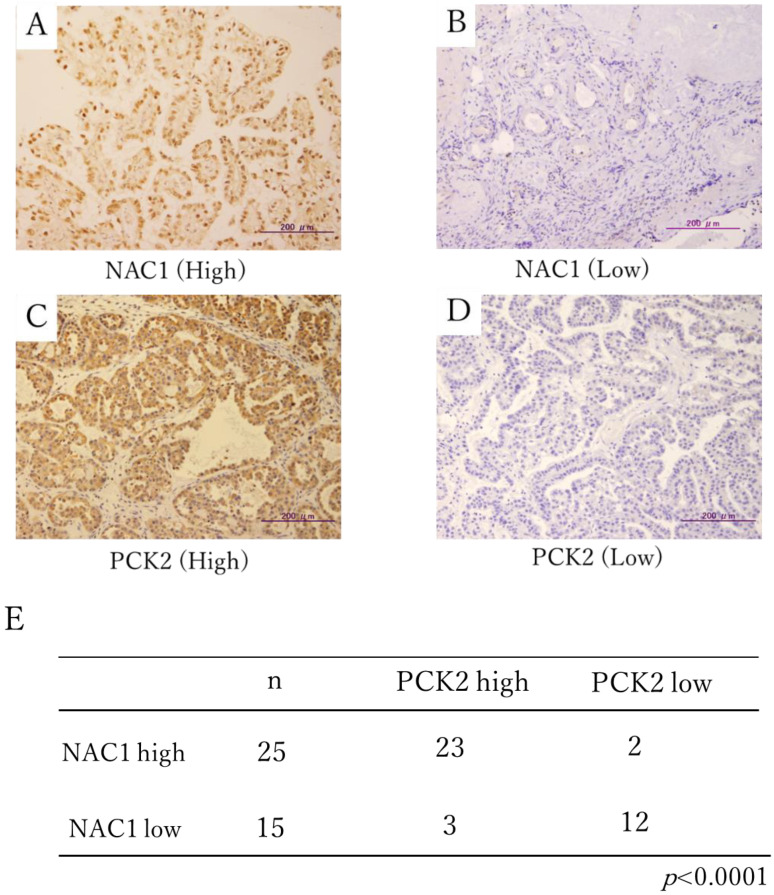
Immunohistochemical staining of NAC1 and PCK2 in ERON surgical specimens. Representative cases with high (**A**) and low (**B**) NAC1 expression and high (**C**) and low (**D**) PCK2 expression are shown. (**E**) Statistical correlation between NAC1 and PCK2 protein expression levels in tumor tissues. Abbreviations: NAC1, nucleus accumbens-associated protein 1; PCK2, phosphoenolpyruvate carboxykinase 2; ERON, endometriosis-related ovarian neoplasm.

Figure 1A–D shows representative immunohistochemical staining of NAC1 and PCK2 in ovarian cancer surgical specimens. NAC1 expression was observed in the nuclei, while PCK2 was detected in the cytoplasm, consistent with its mitochondrial localization.

High NAC1 expression was detected in 63% (25/40) of tumor samples. Among these 25 NAC1-high cases, 23 (92%) also exhibited high PCK2 expression, while only 2 (8%) were PCK2-low. Conversely, among the 15 NAC1-low cases, 12 (80%) demonstrated low PCK2 expression. A significant positive association was observed between NAC1 and PCK2 expression (Chi-square test, χ^2^ = 18.3, *p* < 0.0001; phi coefficient = 0.68; Figure 1E).

### 2.2. Elevated Expression of NAC1 and PCK2 Is Associated with Poorer Overall Survival (OS) in Patients with ERONs

**Figure 2:**
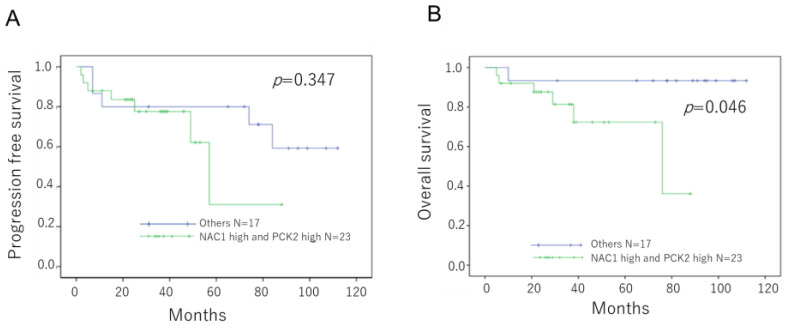
Prognostic significance of NAC1 and PCK2 expressions in patients with ERONs Kaplan–Meier survival curves show PFS (**A**) and OS (**B**) stratified by combined NAC1 and PCK2 expression status. Patients with high expression of both NAC1 and PCK2 exhibited significantly shorter OS. Abbreviations: NAC1, nucleus accumbens-associated protein 1; PCK2, phosphoenolpyruvate carboxykinase 2; ERON, endometriosis-related ovarian neoplasm; OS, overall survival; PFS, progression-free survival.

Patients with ovarian cancer were divided into two groups based on the combined expression status of NAC1 and PCK2: those with high co-expression of both proteins versus those with all other expression patterns. As shown in Figure 2, patients in the NAC1-high/PCK2-high group had significantly shorter OS than those in the other group (*p* = 0.046, log-rank test). Although progression-free survival (PFS) was shorter in the NAC1-high/PCK2-high group, the difference did not reach statistical significance (*p* = 0.345, log-rank test). These findings suggest that co-expression of high NAC1 and PCK2 levels may serve as a prognostic marker in patients with ERONs.

### 2.3. NAC1 Directly Regulates PCK2 Expression

#### 2.3.1. NAC1 Expression Correlates with PCK2 Expression in Gynecologic Cancer Cell Lines

**Figure 3:**
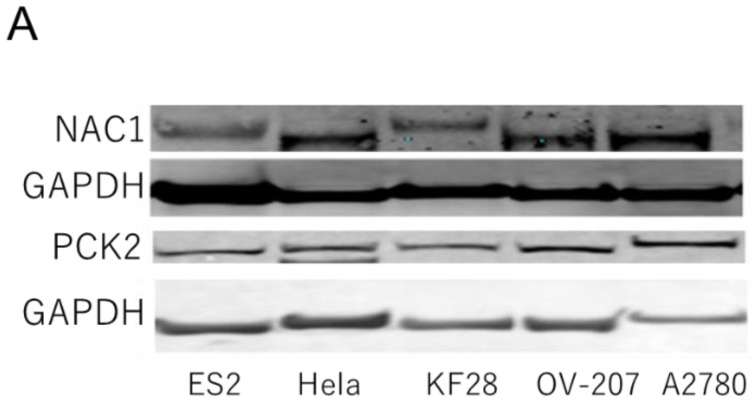

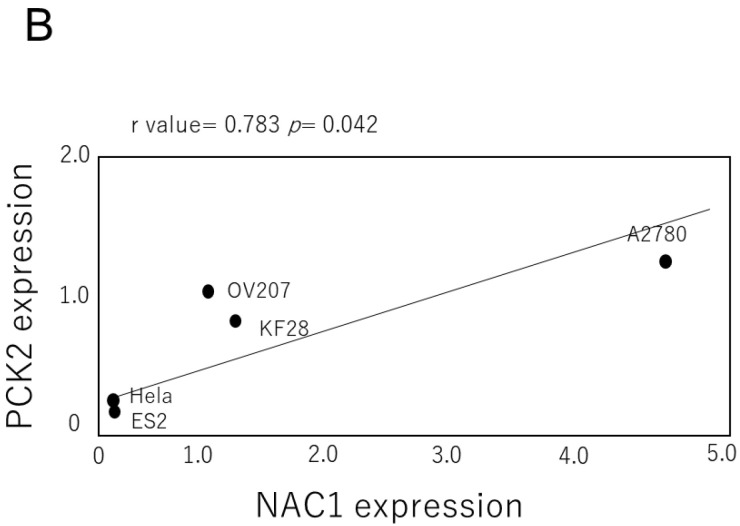
Correlation between NAC1 and PCK2 expression in ovarian cancer cell lines. (**A**) Representative Western blot analysis of NAC1 and PCK2 expression in various ovarian cancer cell lines. (**B**) Quantitative correlation between NAC1 and PCK2 protein levels, evaluated by densitometry using ImageJ software (version 1.53k). Abbreviations: NAC1, nucleus accumbens-associated protein 1; PCK2, phosphoenolpyruvate carboxykinase 2.

NAC1 and PCK2 expression levels were assessed in several gynecological cancer cell lines, including ovarian and cervical cancer cell lines. As shown in Figure 3, NAC1 and PCK2 protein levels were positively correlated in ovarian cancer cells.

**Figure 4:**
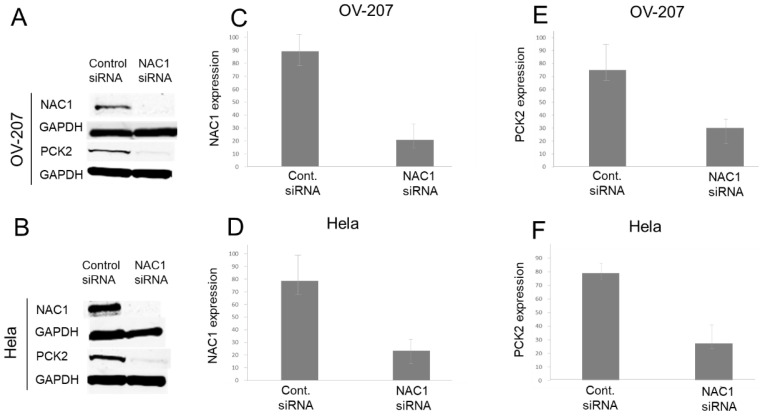
NAC1 knockdown reduces PCK2 expression in gynecologic cancer cell lines. Western blot analysis of NAC1 and PCK2 expression following siRNA-mediated NAC1 knockdown in OV207 (**A**) and HeLa (**B**) cells. (**C**,**D**) Quantification of NAC1 knockdown efficiency. (**E**,**F**) Downregulation of PCK2 expression following NAC1 knockdown. Data confirm that silencing NAC1 significantly reduces PCK2 expression in both cell lines. Abbreviations: NAC1, nucleus accumbens-associated protein 1; PCK2, phosphoenolpyruvate carboxykinase 2; siRNA, small interfering RNA.

To investigate whether NAC1 regulates PCK2 expression, knockdown experiments were conducted in OV207 and HeLa cells, both of which endogenously express NAC1 and PCK2. Silencing of NAC1 using small interfering RNA (siRNA) resulted in a marked reduction in PCK2 protein levels, particularly in OV207 cells (Figure 4). This suggests that NAC1 positively regulates PCK2 expression in gynecologic cancer cells.

#### 2.3.2. NAC1 Activates PCK2 Promoter Activity

**Figure 5:**
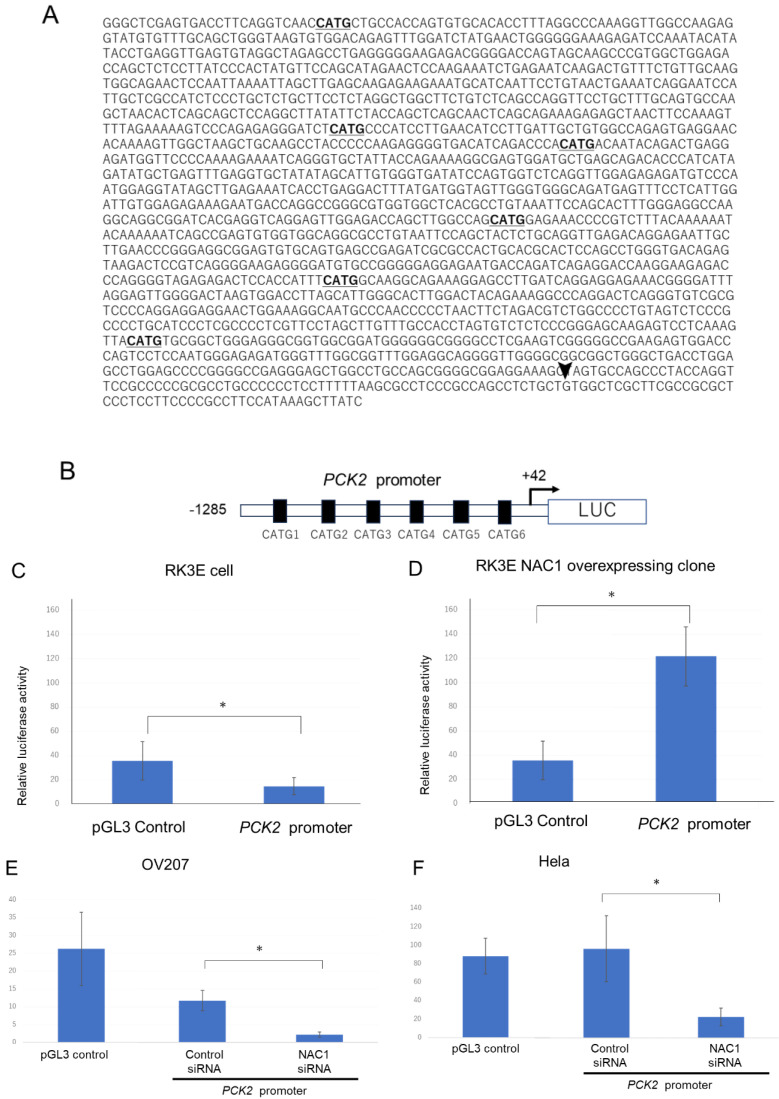
Transcriptional activity of the PCK promoter in ovarian cancer cell lines. (**A**) Schematic representation of the promoter region spanning from −1285 to +42 relative to the transcriptional start site (arrowhead). Underlined sequences indicate six CATG motifs, the consensus binding sites for NAC1. (**B**) The full-length PCK2 promoter was cloned into the pGL3-Basic luciferase reporter vector for promoter activity analysis. (**C**,**D**) Luciferase reporter assays were performed in RK3E cells lacking endogenous NAC1 (**C**) and RK3E cells with stable NAC1 overexpression (**D**). (**E**,**F**) Promoter activity was further evaluated in OV207 and HeLa cells, both of which express endogenous NAC1. NAC1 was silenced using siRNA, and changes in PCK2 promoter activity were measured. * *p* < 0.05. Abbreviations: NAC1, nucleus accumbens-associated protein 1; PCK2, phosphoenolpyruvate carboxykinase 2; LUC, luciferase; siRNA, small interfering RNA.

Next, we investigated the mechanism by which NAC1 regulates PCK2 expression. To determine whether NAC1 activates PCK2 promoter activity, we performed a luciferase reporter assay in ovarian cancer cells. Promoter sequences ranging from +42 to −1285 upstream of the transcriptional start site of the human *PCK2* gene were cloned into luciferase reporter constructs, as illustrated in Figure 5A,B. For the promoter assay, we used RK3E cells, which do not express endogenous NAC1, and OV207 cells, which express endogenous NAC1. Luciferase activity was assessed following transient transfection with the PCK2 promoter construct. The PCK2 promoter activity was induced in RK3E cells stably expressing NAC1 but not in native RK3E cells (Figure 5C,D). Similarly, in OV207 and HeLa cells—which endogenously express NAC1—PCK2 promoter activity was observed and significantly reduced following NAC1 knockdown by siRNA (Figure 5E,F). These findings indicate that human PCK2 promoter activity is regulated by NAC1 expression in ovarian cancer cells.

**Figure 6:**
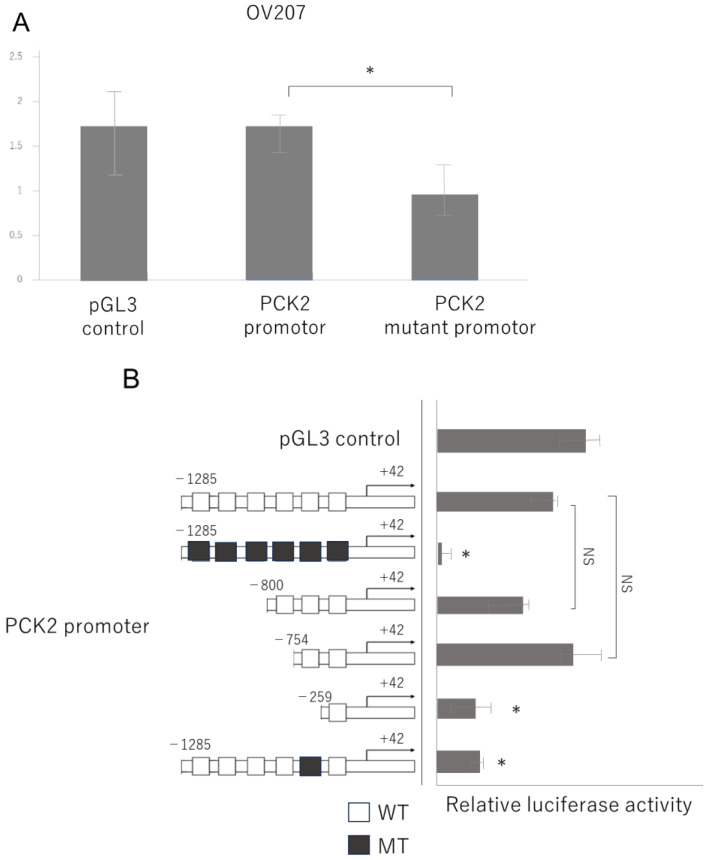
The fifth CATG motif is critical for NAC1-mediated activation of the PCK2 promoter. (**A**) Promoter activity of the *PCK2* gene is significantly reduced in OV207 cells transfected with a construct containing alanine substitutions for all six CATG motifs compared to the wild-type promoter. (**B**) Mutation of the fifth CATG motif alone significantly reduces promoter activity, confirming its key role in NAC1-mediated transcriptional activation. Abbreviations: NAC1, nucleus accumbens-associated protein 1; PCK2, phosphoenolpyruvate carboxykinase 2. *, *p* < 0.01; NS, not significant.

To further assess the functional relevance of PCK2 in ovarian cancer cells, we performed a proliferation assay after PCK2 knockdown by siRNA. As shown in Figure 6, PCK2 knockdown significantly inhibited cell proliferation, suggesting that PCK2 plays a critical role in promoting ovarian cancer cell growth.

### 2.4. NAC1 Binds to the PCK2 Gene Promoter Directly Through Its Recognition Sequence, CATG

#### 2.4.1. The 5th CATG Sequence Is Critical for NAC1-Mediated Promoter Activation

Next, we investigated whether NAC1 activates the PCK2 promoter by binding to it. We previously reported that NAC1 recognizes and binds to the CATG sequence on DNA [13]. A visual search of the PCK2 promoter sequence identified six CATG motifs (Figure 5A, bold). To assess the functional importance of these motifs, we first engineered a mutant promoter in which all CATG motifs were replaced with alanine substitutions and assessed promoter activity using a luciferase assay. Promoter activity was significantly lower in the mutant than in the wild-type promoter (Figure 6A).

To determine which CATG site was responsible for NAC1-mediated promoter activation, we generated a series of PCK2 promoter deletion constructs, as illustrated in Figure 6B. Each construct selectively removed individual CATG sites. Luciferase assays revealed that deletion of the fifth CATG motif markedly impaired promoter activity, suggesting that this specific site is critical for the transcriptional activation of PCK2 by NAC1. Next, to validate the contribution of the fifth CATG motif to promoter activity, we generated a mutant PCK2 promoter construct in which only the fifth CATG was replaced with an alanine substitution. The results demonstrated a more significant reduction in promoter activity than in the wild-type sequence (Figure 6B). These findings suggest that the fifth CATG motif, located approximately 754 base pairs upstream of the transcriptional start site (+1), plays a critical role in PCK2 promoter activation by NAC1.

#### 2.4.2. Chromatin Immunoprecipitation (ChIP) Assay Revealed That Endogenous NAC1 Directly Binds to the Fifth CATG Motif in the PCK2 Promoter in OV207 Cells

**Figure 7:**
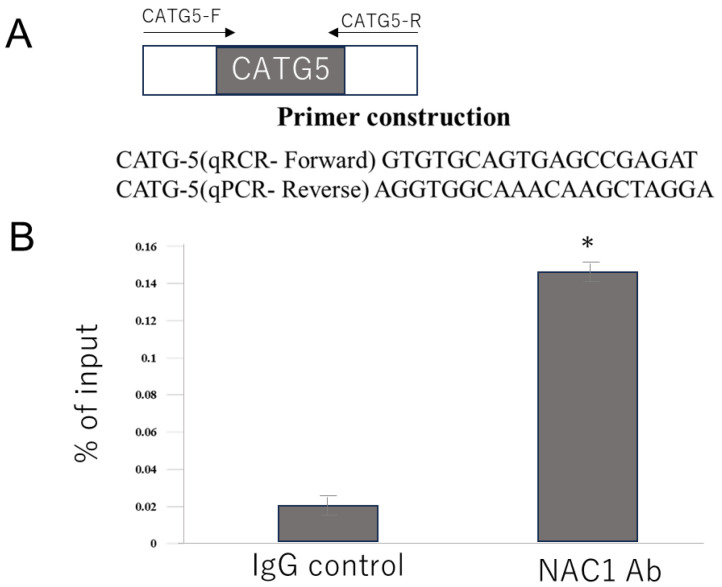
ChIP assay demonstrates NAC1 binding to the fifth CATG site on the PCK2 promoter in OV207 cells. (**A**) Primer design for qPCR targeting the promoter region containing the fifth CATG motif. (**B**) qPCR results showing greater enrichment of the fifth CATG-containing DNA fragment following immunoprecipitation with anti-NAC1 antibody than that with the control IgG, using the primers described above. Data represent mean ± SD from three independent experiments (*n* = 3). * *p* < 0.05. Abbreviations: NAC1, nucleus accumbens-associated protein 1; PCK2, phosphoenolpyruvate carboxykinase 2; qPCR, quantitative polymerase chain reaction; IgG, immunoglobulin G.

To confirm whether NAC1 binds directly to the fifth CATG motif in vivo, a ChIP assay was performed. Primers for quantitative real-time polymerase chain reaction (qPCR) were designed to amplify the DNA region containing the fifth CATG motif (Figure 7A). Chromatin from OV207 cells was immunoprecipitated using an anti-NAC1 antibody or a control IgG. qPCR analysis of the precipitated DNA revealed that the signal obtained with the anti-NAC1 antibody was significantly higher than that with the control antibody (Figure 7B). These findings confirm that NAC1 directly binds to the region of the PCK2 promoter containing the fifth CATG motif in living cells.

### 2.5. PCK2 Knockdown Represses the Viability of Ovarian Cancer Cells

**Figure 8:**
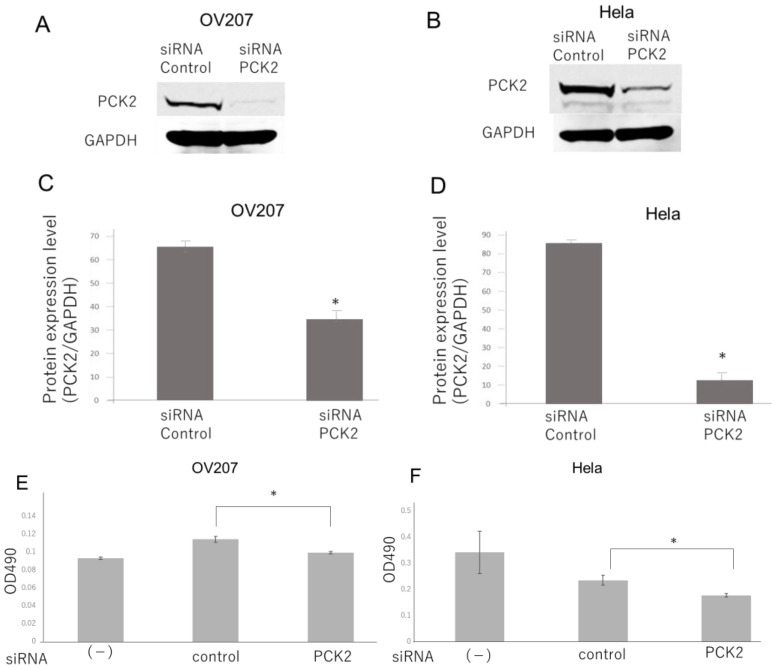
Cell proliferation of OV207 and HeLa cells following PCK2 knockdown ((**A**,**C**); OV207, (**B**,**D**); Hela) was evaluated using the MTT assay, with absorbance measured at OD490. MTT signal was significantly lower in cells transfected with PCK2 siRNA than in those transfected with control siRNA on Day 4 (**E**,**F**). Abbreviations: PCK2, phosphoenolpyruvate carboxykinase 2; siRNA, small interfering RNA; OD490, optical density at 490 nm. *, *p* < 0.01.

To investigate the effects of PCK2 on cell viability and metabolic activity, MTT assays were performed in OV207 and HeLa cells with and without PCK2 knockdown. As shown in Figure 8, the MTT signal was significantly reduced in the PCK2 knockdown group compared with the control group, indicating decreased mitochondrial activity and reduced cell survival. While this assay does not directly measure proliferation, these results suggest that PCK2 influences both cell viability and cellular metabolic activity.

### 2.6. NAC1 Regulates Truncated Gluconeogenesis Through PCK2

**Figure 9:**
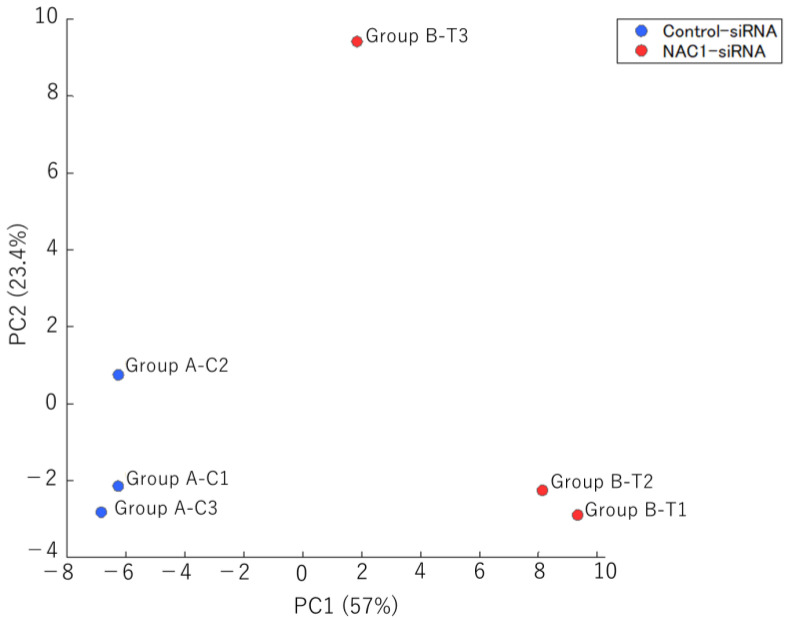
PCA of metabolomic profiles in OV207 cells with and without NAC1 knockdown. PCA clearly distinguished the two groups (*n* = 3 per group), with PC1 capturing variance primarily associated with NAC1 expression status. Abbreviations: PCA, principal component analysis; PC1, principal component 1; PC2, principal component 2; NAC1, nucleus accumbens-associated protein 1.

To determine which metabolic pathway is regulated by NAC1 through PCK2 expression, we analyzed the metabolomic profiles of OV207 cells with and without NAC1 knockdown and compared metabolite levels between the two groups. OV207 cells were cultured, and knockdown assays were performed using NAC1-targeting siRNA and control siRNA. Metabolomic analysis was performed to identify differences in metabolite profiles between NAC1 knockdown and control cells. As shown in Figure 9, principal component analysis (PCA) revealed a clear separation between the two groups along principal component 1 (PC1), which reflected metabolic changes induced by NAC1 knockdown.

**Figure 10:**
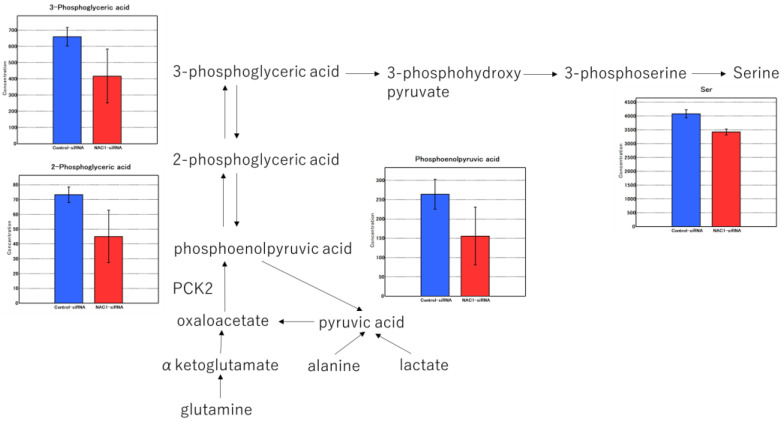
Metabolomic profiles in NAC1 knockdown versus control OV207 cells. Truncated gluconeogenesis was suppressed by NAC1 KD. NAC1 knockdown suppressed truncated gluconeogenesis, as evidenced by significantly lower levels of phosphoenolpyruvic acid, 2-phosphoglyceric acid, 3-phosphoglyceric acid, and serine. The values represent means ± SD. PCK2 catalyzes the conversion of OAA to PEP, a key step in the truncated gluconeogenesis pathway. Abbreviations: NAC1, nucleus accumbens-associated protein 1; KD, knockdown; PCK2, phosphoenolpyruvate carboxykinase 2; OAA, oxaloacetate; PEP, phosphoenolpyruvate; SD, standard deviation.

**Figure 11:**
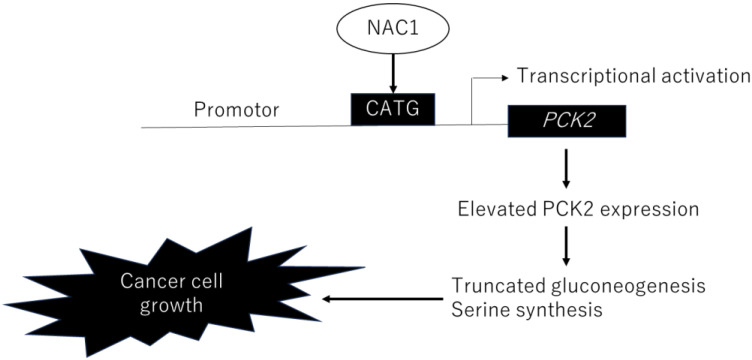
Proposed model illustrating the role of NAC1 in ERON development through the regulation of PCK2 expression. Abbreviations: NAC1, nucleus accumbens-associated protein 1; PCK2, phosphoenolpyruvate carboxykinase 2; ERONs, endometriosis-related ovarian neoplasms.

The factor loadings for PC1 are listed in Table 1. Notably, phosphoenolpyruvic acid, 2-phosphoglyceric acid, 3-phosphoglyceric acid, and serine were major contributors to PC1 (Table 1, red). Consistent with this, the levels of these metabolites were significantly lower in NAC1 knockdown cells than in control cells (*p* < 0.005) (Figure 10). Serine production was also markedly reduced under NAC1 knockdown conditions. Furthermore, malic acid and citric acid—key intermediates in the TCA cycle—as well as lactic acid, the end product of glycolysis, were also found at lower levels in NAC1 knockdown cells. These findings suggest that NAC1 promotes the expression of PCK2 and enhances the production of metabolites involved in a truncated gluconeogenic pathway, particularly phosphoenolpyruvic acid, 2-phosphoglyceric acid, and 3-phosphoglyceric acid. A proposed model summarizing this mechanism is shown in Figure 11.

### 2.7. NAC1–PCK2 Axis Regulates De Novo Serine Synthesis

**Figure 12:**
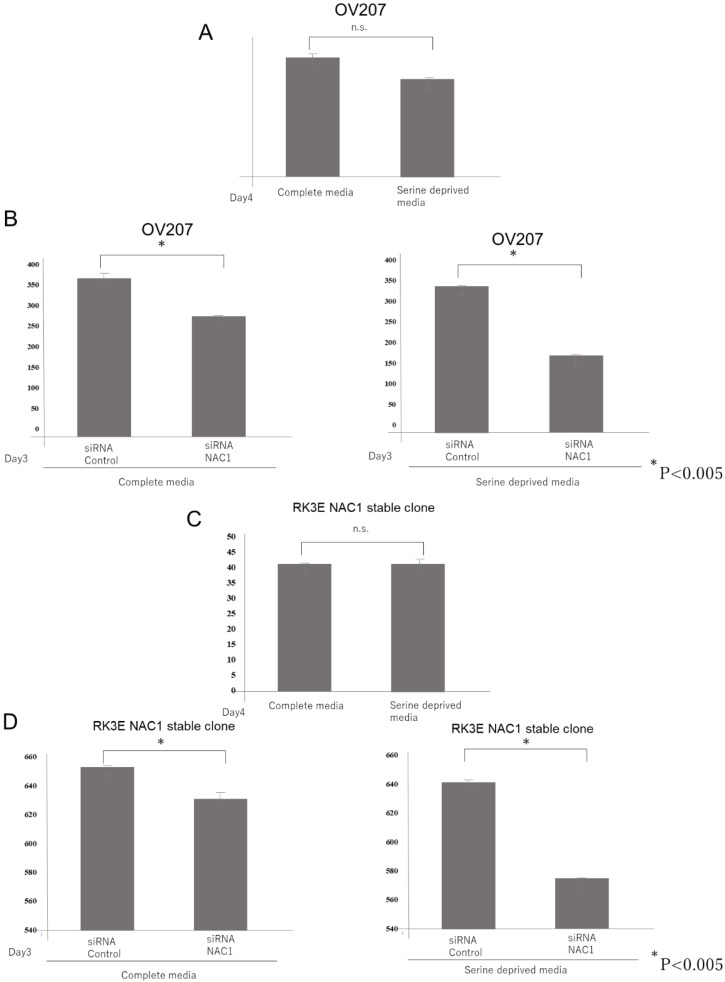
NAC1 knockdown-induced growth suppression is more pronounced in serine-deprived media than in complete media. The growth rate of OV207 cells and NAC1-overexpressing stable clones of NAC1-null RK3E cells did not differ significantly between complete and serine-deprived media (**A**,**C**). Growth suppression by NAC1 Knockdown was more pronounced in serine-deprived media than in complete media (**B**,**D**). Abbreviations: NAC1, nucleus accumbens-associated protein 1; siRNA, small interfering RNA; n.s., not significant.

Notably, we found that serine levels were significantly decreased following NAC1 knockdown. We interpreted this as a consequence of reduced 3-phosphoglyceric acid availability, which serves as a precursor for de novo serine synthesis. Under serine-deprived conditions—or when the cellular demand for serine is elevated—many cancer cells adapt by activating the serine synthesis pathway (SSP). We hypothesized that the NAC1–PCK2 axis enhances serine production by promoting truncated gluconeogenesis, thereby conferring a growth advantage. To test this, we cultured NAC1-expressing OV207 cells in media with and without serine and compared the effects of NAC1 knockdown under both conditions. As shown in Figure 12A, the growth rate of OV207 cells did not differ significantly between complete and serine-deprived media under normal conditions. However, upon NAC1 knockdown using siRNA, growth suppression was more pronounced in serine-deprived media than in complete media (Figure 12B). This assay was replicated using NAC1-overexpressing stable clones of NAC1-null RK3E cells, yielding similar results (Figure 12C,D). These findings suggest that NAC1 promotes cell growth under serine-limited conditions by supporting de novo serine biosynthesis through the PCK2-mediated truncated gluconeogenic pathway.

## 3. Discussion

In this study, we provide new evidence suggesting that the cancer-related transcription factor NAC1 may modulate cellular metabolism in cancer cells by activating the expression of its downstream target gene, PCK2, a key enzyme in gluconeogenesis, in ERONs. We also demonstrated that NAC1 and PCK2 expression levels were highly correlated in surgical specimens from ovarian cancer and that elevated expression of both proteins is associated with poorer prognosis in patients with ERONs.

PCK2 is a rate-limiting enzyme in gluconeogenesis and is located in the mitochondria. It catalyzes the conversion of OAA and guanosine triphosphate to PEP, CO_2_, and guanosine diphosphate and is typically expressed only in gluconeogenic organs, such as the liver and kidney [14]. Recently, PCK2 has been reported to contribute to cancer development [15] and is abnormally expressed in various cancers, including colorectal cancer [16], lung cancer [17], melanoma [18], and prostate cancer [11]. To our knowledge, this is the first study to demonstrate aberrant PCK2 expression in ovarian cancer and to suggest its potential as a prognostic marker and therapeutic target for ERONs.

Proliferating cells have a high demand for glycolytic intermediates, which serve as essential substrates for various biosynthetic pathways, including the synthesis of nucleic acid, lipids, serine, and glutathione. To support these biosynthetic needs, cancer cells reprogram their metabolism to enhance glycolysis and increase the availability of glycolytic intermediates necessary for sustained proliferation [19]. Accordingly, cancer cells exhibit increased glucose uptake and elevated glycolytic activity, while the TCA cycle is relatively repressed—even in the presence of oxygen. The characteristic metabolic shift, known as aerobic glycolysis or the Warburg effect, is a hallmark of many cancers [19]. However, the tumor microenvironment is often nutrient-deprived. As tumors grow, they activate angiogenesis to improve blood supply and ensure adequate glucose delivery. Despite this, the newly formed vasculature is typically leaky and inefficient, often failing to meet the metabolic needs of the growing tumor mass [20]. Consequently, cancer cells frequently experience glucose scarcity. To compensate for this limited glucose availability while maintaining the production of glycolytic intermediates, cancer cells may upregulate gluconeogenesis—the metabolic pathway that generates glucose from non-carbohydrate precursors. PCK2, a rate-limiting enzyme in gluconeogenesis, plays a central role in this adaptive response [21]. ERONs are characterized by a growth pattern that results in the formation of large tumor masses, in contrast to serous ovarian carcinomas, which primarily spread through peritoneal dissemination. The localized growth pattern makes ERONs more susceptible to nutritional deficiencies, underscoring the importance of metabolic pathway adaptation for their sustained growth [22].

We demonstrated that NAC1 upregulates PCK2 expression, thereby enhancing truncated gluconeogenesis in ovarian cancer cells. Our findings also indicate that PCK2 plays a critical role in maintaining cell viability and metabolic activity, highlighting its potential contribution to ovarian cancer cell survival. To date, only a limited number of studies have addressed the regulatory mechanisms controlling PCK2 expression in cancer. For example, Yan Sun et al. reported that the esophageal cancer-related transcriptional regulator PURα (purine-rich element binding protein A) directly increases PCK2 expression by binding to its specific promoter site. This activation induces metabolic changes in esophageal cancer cell lines by modulating mitochondrial respiration and glycolytic capacity [23]. Similarly, in colorectal cancer cells, PGC-1β and estrogen-related receptors enhance glutamine metabolism to promote cell survival by upregulating PCK2 transcription [24]. In another study, Vincent et al. showed that HIF-1α activates PCK2 expression and promotes PEP production from glutamine under glucose-limited conditions in lung cancer cells [25]. This is the first study to clarify the regulatory mechanism of PCK2 expression by NAC1 in ovarian cancer cells. NAC1 was initially identified as a molecule highly expressed in the brain cells of cocaine addicts [26] but was recognized as a transcription factor associated with ovarian cancer progression [6]. NAC1 recognizes the consensus-binding sequence CATG, as previously reported, and directly binds to this motif within the promoter region of PCK2 [13]. Among the six CATG sites identified in the PCK2 promoter, the fifth site is responsible for NAC1 binding. We confirmed that the knockdown of NAC1 resulted in reduced PCK2 expression, supporting the notion that NAC1 functions as a transcriptional activator of PCK2 (Figure 4). This suggests the possibility of a unique regulatory mechanism governing PCK2 expression that may be influenced by the organ specificity of the cancer.

In lung cancer cells, PCK2 enzymatic activity increases under glucose-depleted conditions, promoting the conversion of glutamine to PEP, which subsequently supports the biosynthesis of serine and purines [17]. In this study, we observed that NAC1 also influences serine production. Metabolomic analysis revealed that NAC1 knockdown reduced serine levels in ovarian cancer cells. The de novo serine synthesis pathway is mediated by glycolytic intermediates, suggesting that the NAC1–PCK2 axis may enhance serine biosynthesis via PEP production. However, we did not determine whether the NAC1–PCK2 axis promotes glutamine metabolism to support gluconeogenesis in ovarian cancer cells.

Serine plays a critical role in several biological processes, including protein, nucleotide, and lipid synthesis; glutathione and NADPH generation via glycine; and the donation of one-carbon units for the folate cycle and methylation reactions [27]. As a non-essential amino acid, serine can be obtained either through uptake from the extracellular environment or synthesized de novo via the SSP. Certain cancer cells exhibit high serine demand and rely heavily on exogenous serine for optimal growth [28,29,30,31]. Under serine-deprived conditions or high serine demands, cancer cells can adapt by activating de novo SSP. In some cancers, this adaptation involves amplification or overexpression of PHGDH, the enzyme that catalyzes the first step of the SSP, enabling these cells to better tolerate serine starvation [32]. To assess the contribution of the SSP in ovarian cancer cells, we performed a growth assay under NAC1 knockdown conditions, with and without exogenous serine supplementation. The results revealed that growth inhibition caused by NAC1 knockdown was significantly more pronounced under serine-deprived conditions, mimicking an environment in which exogenous serine supply was restricted (Figure 12). These findings suggest that ovarian cancer cells rely on serine availability for their proliferation and NAC1 supports serine biosynthesis. Therefore, inhibition of NAC1 may exert a synergistic anti-proliferative effect when combined with strategies that limit external serine sources, such as a serine-restricted diet. This study has some limitations. First, it remains unclear which specific metabolic alterations—such as increased glutamine metabolism associated with PCK2 activation in lung cancer cells—also occur during PCK2-mediated enhancement of gluconeogenesis in ovarian cancer cells. This aspect warrants further investigation. Second, given the dynamic nature of metabolic reactions, the reliability of metabolomic analysis based on metabolite levels measured at a single time point is limited. Third, the isotope tracing experiment is absent. Such experiments would be important to directly demonstrate carbon flow from PCK2 activity into the serine synthesis pathway and should be addressed in future investigations. Forth, rescue experiment, such as restoring PCK2 in NAC1-knockdown cells to confirm the dependency of cell growth on this metabolic pathway, is absent. Fifth, although NAC1 and PCK2 expression correlates with overall survival in univariate analysis, this study could not determine whether it is an independent prognostic factor. Future studies with larger patient cohorts and multivariable analysis are required. Sixth, our findings from in vitro experiments were not validated in vivo. Therefore, further studies are required to explore the therapeutic potential of targeting the NAC1–PCK2 axis.

## 4. Materials and Methods

### 4.1. Cell Lines

Human gynecological cancer cell lines, including OV207, HeLa, RK3E, KF28, ES2, and A2780, were obtained from the American Type Culture Collection, Manassas, VA, USA.

Characterization and authentication of the cell lines were performed by the cell banks using morphological assessment, karyotyping, PCR, and short tandem repeat profiling. To maintain authenticity, multiple aliquots of frozen stocks were prepared from the initial stocks, and a fresh frozen aliquot was used every 3 months for experiments. Cells were routinely monitored for identity by morphological evaluation and growth curve analysis, and they were confirmed to be free of mycoplasma contamination.

### 4.2. Tissue Samples

Formalin-fixed, paraffin-embedded (FFPE) surgical samples from 40 patients with ERONs were collected from patients who underwent surgery at the Department of Obstetrics and Gynecology, Shimane University Hospital. Pathological diagnoses were based on standard morphological examination of hematoxylin and eosin-stained sections and classified according to the World Health Organization (WHO) criteria. Tumor staging was performed in accordance with the International Federation of Gynecology and Obstetrics classification. The acquisition of tissue specimens and clinical information was approved by the Institutional Review Board of Shimane University (IRB No. 20070305-2, version 10; last update, 8 December 2019).

### 4.3. Immunohistochemistry

Monoclonal anti-NAC1 (clone 9.27) antibody was used as previously described [8]. For PCK2 detection, a commercially available polyclonal anti-PCK2 antibody (GeneTex Inc., Irvine, CA, USA) was used at a dilution of 1:200.

Immunohistochemical staining was performed on FFPE tissue sections. After deparaffinization and rehydration, the sections were incubated overnight at 4 °C in a humidified chamber with either the anti-NAC1 antibody or the anti-PCK2 antibody. The avidin–biotin–peroxidase complex method was employed, and immunoreactivity was visualized using 3,3′-diaminobenzidine as the chromogen. Expression levels of NAC1 and PCK2 were evaluated using the H-score method. The H-score was calculated by multiplying the percentage of positively stained tumor cells (0–100%) by the staining intensity, graded as follows: 0 = negative, 1 = weak, 2 = moderate, and 3 = strong. This yields a total score ranging from 0 to 300. Based on the H-score, samples were categorized into four groups: 0, 1+, 2+, and 3+. Scores of 0 and 1+ were defined as low expression, whereas scores of 2+ and 3+ were considered high expression. All evaluations were performed in triplicate, and the mean H-score was used for subsequent analyses.

### 4.4. Western Blot

Cells were lysed in lysis buffer, heated at 100 °C for 5 min, and then cooled on ice for 1 min. Lithium dodecyl sulfate sample buffer and a reducing agent were added, followed by centrifugation at 15,000 rpm for 5 min. Duplicate samples containing 10 μg of protein were subjected to sodium dodecyl sulfate–polyacrylamide gel electrophoresis and transferred to polyvinylidene fluoride membranes. Membranes were blocked with 5% skim milk in Tris-buffered saline with 0.1% Tween 20 (TBST) for 1 h at room temperature and then incubated overnight at 4 °C with either anti-NAC1 antibody (1:250, Abcam, Cambridge, UK) or anti-PCK2 antibody (1:700, Sigma-Aldrich, St. Louis, MO, USA) on a shaker. After four washes with TBST, membranes were incubated with horseradish peroxidase (HRP)-conjugated secondary antibodies—either anti-rabbit IgG-HRP (sc-2357, Santa Cruz Biotechnology, Dallas, TX, USA) or anti-mouse IgG-HRP (1:10,000)—for 1 h at room temperature. As a loading control, membranes were probed with anti-GAPDH antibodies (1:10,000, Cell Signaling Technology, Beverly, MA, USA). Protein bands were visualized using the Amersham ImageQuant 800 imaging system (Cytiva, Marlborough, MA, USA). Band intensity and near-infrared fluorescence signals were quantified using ImageQuant 8.1 software. Background signals within each lane were subtracted, and bounding boxes were manually adjusted around each band to ensure accurate quantification.

### 4.5. Silencing RNA Knockdown of NAC1 Gene Expression

Stealth siRNA targeting NAC1 (sequence #1: 5′-CCGGCUGAACUUAUCAACCAGAUUG-3′) [Nishi] was purchased from Thermo Fisher Scientific (Waltham, MA, USA). Cells were seeded in 96-well plates at a density of 8 × 10^5^ cells per well and transfected with siRNAs using Lipofectamine RNAiMAX (Thermo Fisher Scientific) according to the manufacturer’s protocol. After 48 h of incubation, luminescence was measured using a luminometer. Data are presented as the mean ± standard deviation from triplicate measurements.

### 4.6. Luciferase Assay for Promoter Activity

To generate promoter mutants, the CATG motifs recognized by NAC1 were replaced with AAAA sequences to prevent NAC1 binding. The human *PCK2* full-length promoter, a mutant construct with all CATG motifs mutated to alanine, and a construct with only the fifth CATG motif mutated were cloned into the pGL3-Basic vector (Promega, Madison, WI, USA) and synthesized by the Fast Sequence & Molecular Analysis Center. Deletion mutants of the PCK2 promoter were generated by PCR using the full-length promoter as a template and similarly cloned into the pGL3-Basic vector. All PCR-amplified promoter constructs were verified by Sanger sequencing using a 3130 Genetic Analyzer (Thermo Fisher Scientific) to ensure sequence fidelity and the absence of secondary mutations. The pGL3-Control vector (Promega) was used as a positive control.

The luciferase assay was performed using OV207 ovarian cancer cells according to the manufacturer’s protocol. Luciferase activity was measured in cell lysates using the Dual-Luciferase Reporter Assay System (Promega) and a TD-20/20 luminometer (Promega). Each experiment was independently conducted in triplicate.

### 4.7. Chromatin Immunoprecipitation (ChIP) Assay

To generate mouse monoclonal antibodies against NAC1, the region encoding amino acids 284–357 of human NAC1 was expressed as a His-tagged fusion protein in Escherichia coli. Human ovarian OV207 cells were cross-linked with 1% (*v*/*v*) formaldehyde for 10 min at 37 °C and quenched with 0.125 M glycine for 5 min at 37 °C. Cells were then washed with ice-cold phosphate-buffered saline, lysed in radioimmunoprecipitation assay (RIPA) buffer, and subsequently incubated in nuclear lysis buffer (1% SDS, 10 mM EDTA, 50 mM Tris-HCl, pH 8.1) with protease inhibitors for 10 min on ice. Genomic DNA was sheared by sonication using a Branson Sonifier 250 (Emerson, St. Louis, MO, USA). After centrifugation to remove cellular debris and digestion with RNase A, equal amounts of DNA were incubated with 2 µg of anti-NAC1 antibody or control IgG, both pre-bound to anti-rabbit IgG-conjugated magnetic beads (Dynabeads M-280, Thermo Fisher Scientific). Following extensive washing, DNA–protein complexes were eluted. The precipitated DNA was analyzed by quantitative real-time PCR (qPCR) using primer sets targeting CATG motif-containing regions in the PCK2 promoter. All ChIP–qPCR experiments were performed in triplicate using independent biological replicates.

### 4.8. Cell Viability Assay

Cells were seeded in 96-well plates at a density of 3000 cells per well. Cell number was determined indirectly by an MTT assay [33]. Data were expressed as the mean ± 1 SD of triplicate determinations. An MTT cell growth assay was performed 96 h after treating the cells with C75 (Calbiochem, La Jolla, CA, USA) at 10 μM or with DMSO (control). The data were expressed as a percentage of the DMSO control. The mean and SD were obtained from three experiments.

### 4.9. Metabolomic Analysis

Metabolome profiling was conducted by Human Metabolome Technologies, Inc. (HMT, Yamagata, Japan) using the C-Scope platform. OV207 cells were cultured and prepared according to HMT’s standardized protocols. Approximately 1.0–5.0 × 10^6^ cells were used per sample. Prepared cell samples were shipped to HMT, where metabolite extraction, derivatization, and capillary electrophoresis–mass spectrometry were performed. Data processing, normalization, and statistical analyses were carried out by HMT following their proprietary procedures.

### 4.10. Statistical Analysis

PFS and OS were calculated from the date of diagnosis to the date of first relapse or last follow-up. Kaplan–Meier survival curves were generated, and group differences were assessed using the log-rank test. Continuous variables were compared using Student’s *t*-test, while categorical variables were analyzed using Fisher’s exact test. Statistical significance was set at *p* < 0.05. All statistical analyses were performed using SPSS software (version 21; IBM, Armonk, NY, USA). Protein expression levels were quantified by measuring band intensities from Western blots using ImageJ software (NIH, Bethesda, MD, USA).

## 5. Conclusions

We identified NAC1 as a direct transcriptional activator of PCK2, demonstrating that NAC1 modulates cancer metabolism by enhancing truncated gluconeogenesis and de novo serine synthesis in ERONs. PCK2 plays a critical role in maintaining cell viability and metabolic activity. These findings suggest that the NAC1–PCK2 axis may serve both as a prognostic marker and as a promising therapeutic target in ovarian cancer.

## Figures and Tables

**Table 1:** Top and bottom 30 metabolites based on factor loadings.

Metabolites (Bottom 30)	Factor Loadings	Metabolites (Top 30)	Factor Loadings
IMP	−1.3 × 10^−1^	Citrulline	1.3 × 10^−1^
AMP	−1.3 × 10^−1^	Cys	1.3 × 10^−1^
Adenylosuccinic acid	−1.3 × 10^−1^	Adenosine	1.3 × 10^−1^
Lactic acid	−1.3 × 10^−1^	Asn	1.3 × 10^−1^
Malic acid	−1.3 × 10^−1^	Pro	1.2 × 10^−1^
PRPP	−1.3 × 10^−1^	Acetyl CoA	1.2 × 10^−1^
Citric acid	−1.3 × 10^−1^	Thr	1.2 × 10^−1^
*S*-Adenosylhomocysteine	−1.3 × 10^−1^	cAMP	1.2 × 10^−1^
XMP	−1.3 × 10^−1^	Dihydroxyacetone phosphate	1.2 × 10^−1^
ADP	−1.3 × 10^−1^	Val	1.2 × 10^−1^
GDP	−1.3 × 10^−1^	Succinic acid	1.2 × 10^−1^
2-Phosphoglyceric acid	−1.3 × 10^−1^	Ile	1.2 × 10^−1^
2,3-Diphosphoglyceric acid	−1.2 × 10^−1^	Glu	1.2 × 10^−1^
Ser	−1.2 × 10^−1^	2-Oxoisovaleric acid	1.2 × 10^−1^
3-Phosphoglyceric acid	−1.2 × 10^−1^	Ornithine	1.2 × 10^−1^
*N*-Carbamoylaspartic acid	−1.2 × 10^−1^	His	1.2 × 10^−1^
ADP-ribose	−1.2 × 10^−1^	Fructose 6-phosphate	1.2 × 10^−1^
2-Hydroxyglutaric acid	−1.2 × 10^−1^	Phe	1.2 × 10^−1^
*cis*-Aconitic acid	−1.2 × 10^−1^	Trp	1.2 × 10^−1^
Phosphoenolpyruvic acid	−1.2 × 10^−1^	Ribulose 5-phosphate	1.2 × 10^−1^
GMP	−1.2 × 10^−1^	Leu	1.2 × 10^−1^
Creatine	−1.2 × 10^−1^	Ribose 1-phosphate	1.1 × 10^−1^
NADH	−1.2 × 10^−1^	Gly	1.1 × 10^−1^
NAD^+^	−1.2 × 10^−1^	Met	1.1 × 10^−1^
Xanthine	−1.1 × 10^−1^	Creatinine	1.1 × 10^−1^
NADPH	−1.0 × 10^−1^	Tyr	1.1 × 10^−1^
HMG CoA	−1.0 × 10^−1^	Asp	1.1 × 10^−1^
Argininosuccinic acid	−1.0 × 10^−1^	Glyceraldehyde 3-phosphate	1.1 × 10^−1^
Glutathione (GSSG)	−1.0 × 10^−1^	Glutathione (GSH)	1.1 × 10^−1^
Hypoxanthine	−8.9 × 10^−2^	Putrescine	1.1 × 10^−1^

Metabolites with the highest and lowest loadings for principal component 1 (PC1) are listed. The bottom 30 metabolites include key intermediates of the truncated gluconeogenic pathway, such as phosphoenolpyruvic acid, 2-phosphoglyceric acid, 3-phosphoglyceric acid, and serine. Abbreviations: IMP, inosine monophosphate; AMP, adenosine monophosphate; PRPP, phosphoribosyl pyrophosphate; XMP, xanthosine monophosphate; ADP, adenosine diphosphate; GDP, guanosine diphosphate; Ser, serine; ADP-ribose, adenosine diphosphate ribose; GMP, guanosine monophosphate; NADH, nicotinamide adenine dinucleotide (reduced form); NAD^+^, nicotinamide adenine dinucleotide (oxidized form); NADPH, nicotinamide adenine dinucleotide phosphate (reduced form); HMG CoA, 3-hydroxy-3-methylglutaryl-coenzyme A; GSSG, oxidized glutathione; GSH, reduced glutathione; Cys, cysteine; Asn, asparagine; Pro, proline; Thr, threonine; cAMP, cyclic adenosine monophosphate; Val, valine; Ile, isoleucine; Glu, glutamate; Phe, phenylalanine; Ornithine, ornithine; His, histidine; Trp, tryptophan; Leu, leucine; Gly, glycine; Met, methionine; Tyr, tyrosine; Asp, aspartate.

## Data Availability

The data presented in this study are available on request from the corresponding author (N.N. and K.N.).

## Abbreviations

The following abbreviations are used in this manuscript:

ChIP	chromatin immunoprecipitation
ERON	endometriosis-related ovarian neoplasm
FFPE	formalin-fixed paraffin-embedded
GAPDH	glyceraldehyde-3-phosphate dehydrogenase
GSSG	glutathione disulfide
GSH	glutathione (reduced)
HRP	horseradish peroxidase
NAC1	nucleus accumbens-associated protein 1
OAA	oxaloacetic acid
OS	overall survival
PCA	principal component analysis
PC1	principal component 1
PCK2	phosphoenolpyruvate carboxykinase 2
PEP	phosphoenolpyruvate
PFS	progression-free survival
qPCR	quantitative real-time polymerase chain reaction
RIPA	radioimmunoprecipitation assay
siRNA	small interfering RNA
SSP	serine synthesis pathway
TBST	Tris-buffered saline with Tween 20
TCA	tricarboxylic acid
WHO	World Health Organization
